# Acoustic stimulation in pain management: neurobiological mechanisms and clinical applications—a narrative review

**DOI:** 10.3389/fnins.2026.1861265

**Published:** 2026-06-19

**Authors:** Jianbo Song, Heng Zhang, Linzhi Wu, Zhengjin Duan, Ge Luo, Meihui Li, Lin He

**Affiliations:** 1 Department of Anesthesiology, Mianzhu People’s Hospital, Mianzhu, Sichuan, China; 2 Department of Anesthesiology, Panzhihua Municipal Central Hospital, Panzhihua, Sichuan, China; 3 Department of Traditional Chinese Medicine, People’s Hospital of Fengjie, Fengjie, Chongqing, China; 4 Department of Anesthesiology, The Second Affiliated Hospital of Zhejiang University School of Medicine, Hangzhou, Zhejiang, China

**Keywords:** acoustic stimulation, multimodal analgesic strategies, neurobiological mechanisms, neuromodulation, non-invasive therapy, pain management

## Abstract

Pain affects over 30% of the global population, with an underlying pathogenesis involving a complex interplay of biopsychosocial factors. Despite the availability of conventional pharmacological and interventional therapies, their clinical utility is frequently constrained by concerns regarding substance misuse, surgical complications, and other adverse sequelae. Acoustic stimulation (AS) has emerged as a promising alternative in pain management, characterized by its non-invasive nature, favorable safety profile, and high cost-effectiveness. However, current literature lacks a comprehensive evaluation of cutting-edge AS technologies and a profound decryption of its pleiotropic analgesic mechanisms, which has hindered the integration of AS into multimodal analgesic strategies. This review provides the first comprehensive synthesis of the neurobiological mechanisms and clinical applications of AS. We comprehensively evaluate the analgesic efficacy of diverse modalities, ranging from established interventions—such as music therapy (MT), natural sounds (NS)/noise, and auditory beat stimulation (ABS)—to emerging approaches, including vibroacoustic therapy (VAT) and immersive interactive technologies that integrate multisensory acoustic information. Emerging evidence suggests that AS exerts its therapeutic effects via a multidimensional neurobiological framework, notably through the modulation of corticothalamic circuits, the activation of descending pain-inhibitory systems, and the dynamic regulation of neurochemical mediators. Clinical data consistently highlight the adjunctive value of MT, NS/noise, and VAT in the management of both acute and chronic pain. Furthermore, the convergence of AS with immersive interactive technologies is pioneering a novel digital intervention paradigm, facilitating the seamless integration of AS into multimodal analgesic frameworks. Collectively, these findings suggest that AS represents a robust, non-pharmacological strategy that warrants further exploration as a cornerstone of future personalized, multimodal pain management.

## 1 Introduction

Pain is defined as an inherently subjective sensory and emotional experience, the pathogenesis of which involves a complex interplay of biopsychosocial dimensions (Raja et al., 2020). Current epidemiological data indicate that over 30% of the global population is burdened by chronic pain, posing severe challenges to their quality of life and overall psychological wellbeing (Cohen et al., 2021). Despite the widespread use of conventional pharmacological interventions, such as opioids and non-steroidal anti-inflammatory drugs (NSAIDs), their clinical utility is frequently compromised by risks of substance misuse, dependence, and adverse systemic reactions. Similarly, interventional therapies—including spinal cord stimulation (SCS)—are often constrained by potential surgical complications, risk of infection, and substantial economic burdens (Cohen et al., 2021; Wang and Doan, 2024). Consequently, there is an urgent clinical imperative to develop alternative therapeutic strategies that are non-invasive, safe, and cost-effective.

In recent years, acoustic stimulation (AS) has demonstrated promising therapeutic potential in pain management, owing to its non-invasive nature, cost-effectiveness, and superior safety profile. However, existing literature remains predominantly focused on conventional modalities such as music therapy (MT) and natural sounds (NS). There is a notable lack of a comprehensive evaluation regarding the clinical implementation of emerging frontier technologies, including auditory beat stimulation (ABS), vibroacoustic therapy (VAT), and immersive interactive interventions that integrate acoustic information (Ding S. et al., 2025; Honzel et al., 2019; Koelsch and Bradt, 2025; Shi and Wu, 2023). Furthermore, the discourse surrounding the analgesic mechanisms of AS has long been restricted to psychological interpretations, lacking a profound, multi-layered exploration across neurobiological, cognitive, and multisensory dimensions. These critical knowledge gaps have significantly hindered the transition of AS from a “complementary intervention” toward a core component of multimodal analgesic strategies. Consequently, the clinical translation and technological innovation of advanced AS protocols remain constrained, necessitating a rigorous evaluation of the current evidence to bridge these theoretical and practical divides.

This review provides a comprehensive and up-to-date synthesis of AS in pain management. To ensure a representative and reliable overview, literature selection was guided by a structured search of the PubMed and Web of Science databases for peer-reviewed, English-language articles published over the past decade. The retrieval strategy was meticulously constructed around Medical Subject Headings (MeSH) and core keywords associated with “acoustic stimulation” (encompassing music therapy, binaural beats, vibroacoustic therapy, as well as immersive interactive interventions that integrate acoustic information) and “pain management.” Eligibility criteria focused on clinical trials and observational studies utilizing specified acoustic protocols across all populations, irrespective of age, geographical location, or ethnicity; additionally, landmark basic research studies clarifying the underlying neurobiological mechanisms were also integrated. By conducting a multidimensional analysis of these multifaceted evidence bases, this work seeks to offer critical insights that will facilitate the integration of AS into multimodal analgesic strategies and provide an evidence-based roadmap for future clinical research and technological innovation.

## 2 Overview of acoustic stimulation

Although AS exhibits substantial potential in pain management, a standardized definition remains elusive in academic discourse. Traditionally, AS has been conceptualized as any auditory stimulus capable of eliciting physiological responses (Ding S. et al., 2025). Recent research, however, has underscored the inherent analgesic efficacy of AS (Zhou et al., 2022). With the emergence of multisensory stimulation paradigms and advances in computer-mediated interactive technologies, researchers are increasingly recognizing that relying solely on the auditory pathway may overlook the physical properties of acoustic waves. Integrating acoustic signals with somatosensory and visual modalities allows for more profound neuro-modulation and cortical reorganization, thereby facilitating the exploration of multidimensional, compound analgesic strategies.

Based on the aforementioned theoretical framework, this review defines AS as a non-pharmacological, non-invasive neuromodulatory technique that utilizes acoustic signals with specific physical characteristics (e.g., frequency, beat pattern, and intensity) to act upon the body via auditory or somatosensory pathways, either in unimodal or multimodal forms. The scope of this review encompasses classical acoustic interventions, such as MT, NS, and noise, as well as emerging neuro-acoustic technologies rooted in brainwave entrainment, such as ABS and VAT. Furthermore, although immersive interactive technologies incorporating acoustic information may not strictly conform to the traditional definition of AS, they are included in this analysis as a novel digital intervention paradigm for multimodal analgesia, given their reliance on spatial acoustic information to achieve multisensory synergistic pain relief.

## 3 Neurobiological mechanisms

### 3.1 Multidimensional modulation of the central nervous system (CNS)

Neuroimaging evidence have demonstrated that pain processing is intricately linked to the CNS modulation within complex brain networks, with core regions likely involving the anterior cingulate cortex (ACC), thalamus, prefrontal cortex (PFC), and the periaqueductal gray (PAG) (Shi and Wu, 2023). Consistently, fMRI findings indicate that AS can activate the endogenous nociceptive inhibitory system by enhancing activity in key descending pain-inhibitory regions, including the PAG and rostral ventromedial medulla (RVM), thereby initiating top-down pain suppression pathways at the spinal cord level (Honzel et al., 2019; Shi and Wu, 2023). Basic research has further validated that AS modulates the transmission of nociceptive signals through cortico-thalamic circuits (Zhou et al., 2022).

Furthermore, pain signaling pathways involve the targeted modulation of various neurotransmitters. Research indicates that AS can modulate the release of endogenous opioids, thereby eliciting morphine-like analgesic effects (Chanda and Levitin, 2013; Shi and Wu, 2023). Simultaneously, AS is hypothesized to activate the brain’s rewards system through multisensory stimulation, promoting dopamine secretion, which in turn antagonizes pain perception within higher emotional and cognitive regions such as the limbic system (Honzel et al., 2019; Koelsch and Bradt, 2025; Shi and Wu, 2023). At the cognitive level, the distraction effect of AS is considered to attenuate pain-related cognitive processing, thereby alleviating the patient’s subjective pain experience (Teh et al., 2024). This effect is supported by neurophysiological evidence, particularly in the phenomenon of brainwave synchronization. Electroencephalography (EEG) studies have demonstrated that ABS can influence cortical neuronal excitability through brainwave entrainment, inducing synchronization of alpha or theta oscillations in the cerebral cortex, ultimately achieving pain and anxiety relief (Dos Anjos et al., 2024; Garcia-Argibay et al., 2019; Schmid et al., 2020).

### 3.2 Homeostatic remodeling of the autonomous nervous system (ANS)

Persistent pain frequently triggers a maladaptive state of sympathetic hyperactivation. Extensive clinical trials and neurophysiological studies have demonstrated that AS can effectively modulate autonomic functions by suppressing sympathetic excitability and enhancing vagal tone. This autonomic recalibration induces a state of profound relaxation, thereby mitigating the systemic physiological stress response associated with chronic pain and facilitating the restoration of autonomic homeostasis (Abed et al., 2025; Baltaci et al., 2024; Boyd-Brewer and McCaffrey, 2004; Fleckenstein et al., 2025; Koelsch and Bradt, 2025; Park et al., 2019).

### 3.3 Modulation of inflammatory response and microcirculation

Clinical observational studies have established a strong correlation between chronic pain and elevated levels of pro-inflammatory cytokines. The dysregulation of these inflammatory mediators may even precipitate emotional disturbances, thereby exacerbating the patient’s perceived pain intensity (Jiang et al., 2025). Based on this pathophysiological mechanism, the low-frequency physical vibrations generated by VAT—for instance, at an effective frequency of 40 Hz—may induce localized or systemic tissue resonance. This mechanism is hypothesized to enhance systemic circulation and attenuate inflammatory responses, thereby facilitating pain relief (Ahn et al., 2024).

### 3.4 Targeted modulation of the endocrine system

AS also exerts significant regulatory effects on the endocrine system. Research has demonstrated that AS can downregulate the systemic levels of cortisol and catecholamines, effectively blocking pain hypersensitivity induced by hyperactivation of the hypothalamic-pituitary-adrenal (HPA) axis, AS contributes to the maintenance of systemic pain homeostasis (Chanda and Levitin, 2013; Muzzi et al., 2021).

In summary, the analgesic efficacy of AS is not governed by a singular pathway, but rather by the sophisticated integration of neuro-endocrine-immune networks. This cross-system, multidimensional, and multi-target neurobiological mechanism operates through the synergy of neural functions, psychological cognition, and multisensory experiences. By engaging these diverse regulatory channels, AS facilitates a holistic modulation that ultimately restores both physiological and psychological homeostasis in patients suffering from pain.

## 4 Clinical applications of acoustic stimulation in pain management

### 4.1 Music therapy (MT)

Music therapy is defined as a non-pharmacological intervention utilizing specific physical acoustic properties, such as rhythm, tempo, and harmonic structure. It is primarily categorized into active music therapy, facilitated by professional therapists, and receptive music therapy, which involves the use of patient-preferred music or standardized instrumental compositions within controlled acoustic environments. Extensive evidence-based studies have demonstrated the significant analgesic efficacy of MT across both acute and chronic pain spectra (Arnold et al., 2024; Bradt et al., 2024; Ding S. et al., 2025; Shi and Wu, 2023; Zhou et al., 2022). In the context of acute pain, MT has been shown to elevate pain thresholds and substantially alleviate the negative affective experiences associated with invasive procedures, such as breast biopsies and interventional therapies, with particularly pronounced efficacy in patients exhibiting catastrophic thinking (Chai et al., 2020; Fleckenstein et al., 2025; Soo et al., 2016). With the increasing adoption of Enhanced Recovery After Surgery (ERAS), non-pharmacological perioperative interventions have gained significant clinical recognition. In pediatric populations, MT has been validated to attenuate acute pain during procedures such as heel-stick sampling and tonsillectomy (Chai et al., 2020; Muzzi et al., 2021). Notably, multicenter randomized controlled trials (RCTs) indicate that patients receiving intraoperative therapeutic suggestion accompanied by 20 min of soothing background music exhibited a 24.5% reduction in median opioid consumption within the first 24 postoperative hours compared with the control group (4.0 vs. 5.3 mg; *P* = 0.002), alongside a 25% decrease in mean NRS pain scores (Nowak et al., 2020). These findings suggest that MT possesses a significant opioid-sparing effect, functioning as a potential adjunct or alternative in analgesic strategies.

Regarding chronic pain, the efficacy of MT has been substantiated in conditions including cancer-related pain, fibromyalgia, and low back pain. A randomized controlled trial by Bradt et al. (2024) involving 92 patients with advanced cancer identified pain-related self-efficacy as a core mediator through which MT reduces both pain intensity and pain interference, with indirect effect sizes (ab) of 0.79 (95% CI: 0.01–1.82) and 1.16 (95% CI: 0.02–2.51), respectively. This mechanism subsequently contributed to a reduction in pain-related disability, an effect most prominent in patients with higher baseline pain severity. Thus, pain-related self-efficacy may represent a more critical therapeutic target than emotional relief alone in MT-based chronic pain management. Furthermore, an RCT involving 37 patients with fibromyalgia demonstrated that standardized relaxation music integrated with natural sounds yielded significant analgesic effects persisting for up to 14 days (Alparslan et al., 2016). Additionally, Torlak et al. (2025) reported that a multimodal analgesic approach combining classical music with conventional physical therapy significantly improved Visual Analog Scale (VAS) scores, Beck Anxiety Inventory (BAI) scores, and Neck Disability Index (NDI) scores in patients with chronic neck pain (*P* < 0.05). Nevertheless, systematic evaluations of MT’s efficacy in neuropathic pain and migraines remain limited, necessitating further rigorous clinical validation (Arnold et al., 2024; Sihvonen et al., 2022).

Parallel to the evolution of personalized and precision medicine paradigms, the research focus on MT has progressively shifted from simple efficacy validation toward comparative analyses of varied intervention parameters and musical genres. Indeed, the analgesic outcomes of MT are modulated by multiple critical variables, including individual traits, cultural background, socioeconomic status, and treatment duration. The neurobiological substrate of this “individualized” effect is purportedly linked to the activation of the mesolimbic rewards system and the subsequent release of dopamine (Becker et al., 2025; Cheng et al., 2017; Van der Valk Bouman et al., 2024). Consequently, the clinical implementation of patient-preferred, customized music is likely to yield superior analgesic efficacy.

### 4.2 Natural sounds (NS)/noise

In contrast to the structured rhythms and melodic patterns of MT, NS originate from stochastic acoustic signals within ecological systems, encompassing elements such as the sounds of water, wind, rain, and birdsong (Abed et al., 2025; Baltaci et al., 2024; Buxton et al., 2021). A salient feature of NS in pain management is its distinct temporal dynamics, characterized by a latent and cumulative analgesic efficacy (Zarei et al., 2025). A single-blind RCT involving 60 patients in a burn intensive care unit demonstrated that while a single auditory intervention was insufficient in the early stages of acute trauma, a 7-day NS intervention significantly mitigated patients’ Burn Specific Pain Anxiety Scale (BSPAS) scores and State-Trait Anxiety Inventory (STAI) scores (*P* < 0.001) (Kutenai et al., 2023). In the perioperative care, the implementation of NS during labor significantly improved Apgar scores of neonates at 1 min postpartum [9.04 (SD = 0.815) vs. 8.46 (SD = 1.005), *P* = 0.003], with a significant difference in the recovery trajectory of scores within 5 min (*P* = 0.01) (Parandavar et al., 2025). These findings suggest that perinatal NS may alleviate maternal pain, thereby optimizing neonatal outcomes during the critical early transition period.

In the management of chronic pain, NS is noted for its biological affinity and minimal cognitive load, which typically translates into superior patient adherence. The primary neurophysiological mechanism underlying this effect appears rooted in the modulation of the ANS. Specifically, in patients with fibromyalgia, research by Lepping et al. (2022) indicated that NS—relative to MT—elicits a more robust vagal response and enhances heart rate variability (HRV), thereby facilitating analgesia. Furthermore, NS exhibits a unique capacity to address the comorbidities of chronic pain. Protractedly applied NS can promote deep cortical relaxation and stress reduction, effectively disrupting the “anxiety-pain-insomnia” pathological triad (Buxton et al., 2021). Despite these promising findings, current clinical evidence predominantly situates NS as an adjunctive therapy. The paucity of high-quality, large-scale studies investigating its independent analgesic potency remains a significant barrier to its standardized implementation in clinical protocols.

From a spectral perspective, NS and noise share similar physical acoustic signatures. However, noise primarily functions as a physical masking tool, with its analgesic efficacy largely determined by its spectral power distribution. Emerging evidence suggests that the analgesic potential of acoustic stimuli may be inversely correlated with the signal-to-noise ratio (SNR) (Zhou et al., 2022). Among various noise types, white noise—characterized by a constant power spectral density across the human audible range of 20–20,000 Hz—is the most extensively utilized in clinical settings. Mechanistically, white noise acts by masking deleterious environmental acoustic interference, thereby elevating the pain threshold and reducing sensitivity to nociceptive stimuli (Alkaya Karagoz and Altundogan, 2025; Kolhe et al., 2024; Zhang et al., 2024; Zhou et al., 2022). In pediatric populations, white noise has demonstrated a distinct advantage in providing immediate analgesia during acute procedures. Nevertheless, the application of noise stimulation in adult chronic pain management remains a subject of ongoing debate. This controversy stems from the profound physiological and psychological disparities between adult and pediatric cohorts, particularly regarding chronic pain mechanisms, maladaptive neuroplasticity, and cognitive processing (Alkaya Karagoz and Altundogan, 2025; Kahraman et al., 2020; Kolhe et al., 2024; Lin et al., 2024). Future investigations should prioritize stratified analyses across diverse age cohorts and specific pain subtypes to rigorously evaluate the dose-response relationships of various acoustic spectra. Furthermore, a comprehensive assessment of their clinical efficacy and safety profiles is essential to establish standardized protocols for the implementation of noise-based interventions in multimodal pain management.

### 4.3 Auditory beat stimulation (ABS)

#### 4.3.1 Binaural beats (BB)

Unlike traditional auditory processing pathways, the therapeutic efficacy of BB is primarily mediated by brainwave entrainment. This phenomenon occurs when the brain integrates two slightly different frequencies presented to each ear, leading to neural synchrony in the CNS. Evidence-based data suggest that acoustic-induced synchrony in low-frequency bands—specifically α, θ, and δ waves—promotes profound neural relaxation and exerts significant analgesic effects (Garcia-Argibay et al., 2019; Schmid et al., 2020; Xiong et al., 2025). Among these, 10 Hz α-wave synchrony has demonstrated particular efficacy in modulating acute pain (Ecsy et al., 2017; Maddison et al., 2023).

In perioperative pain management, the clinical utility of BB appears remarkably robust, maintaining its efficacy regardless of anesthetic protocols, intervention timing, or the presence of concurrent background music (Garcia-Argibay et al., 2019; Jang and Choi, 2022; Loong et al., 2022; Ölçücü et al., 2021; Shukla et al., 2025). Notably, BB exhibits a potential pharmacodynamic synergy, enabling a reduction in the dosages of anesthetic agents such as propofol and morphine, which underscores its significant potential in anesthetic management. In pediatric procedural sedation, a RCT involving 49 children undergoing lower abdominal surgery demonstrated that, based on Bispectral Index (BIS) monitoring, perioperative BB intervention (targeting relaxation/sleep frequency bands) reduced the mean propofol infusion rate by 28.6% [3.0 (95% CI: 2.4–3.6) mg⋅kg^–^⋅h^–1^ vs. 4.2 (95% CI: 3.6–4.8) mg⋅kg^–^⋅h^–1^; *P* < 0.01]. Furthermore, in postoperative analgesia for elderly patients, an RCT of 40 individuals undergoing total knee arthroplasty revealed that preoperative 4 Hz BB intervention reduced cumulative morphine consumption at 24 h post-surgery by approximately 50%. These findings highlight the clinical utility of BB as a pharmacological alternative within ERAS protocols, offering a strategy to mitigate opioid-related adverse effects (Schmid et al., 2020; Tani et al., 2021).

Currently, the application of BB in chronic pain is undergoing a critical transition toward clinical translation. A randomized, double-blind, crossover trial by Halpin et al. among patients with chronic pain syndrome demonstrated that 10 Hz α-wave sensory stimulation significantly improved nocturnal pain scores [between-group difference: −0.53 (95% CI: −0.81 to −0.25); *P* < 0.001] and subjective sleep quality [between-group difference: +0.39 (95% CI: 0.15 to 0.64); *P* = 0.002], suggesting that the therapeutic efficacy may be time-dependent (Halpin et al., 2025). Furthermore, while θ-wave synchrony has shown therapeutic potential in chronic pain management, empirical evidence supporting their clinical utility remains insufficient (Ecsy et al., 2017; Garcia-Argibay et al., 2019; Gkolias et al., 2020; Maddison et al., 2023). In summary, as an emerging non-pharmacological and non-invasive neuromodulation strategy, BB has established a preliminary role in the multimodal management of both acute and chronic pain. However, existing literature is largely characterized by small sample sizes and low-level evidence. Future research must prioritize high-quality controlled trials to identify optimal brainwave entrainment protocols tailored to specific pain subtypes.

### 4.4 Vibroacoustic therapy (VAT)

The core mechanism of VAT involves the transduction of acoustic signals into somatosensory inputs, whereby low-frequency sound waves induce resonance in specific bodily regions, potentially activating endogenous reparative processes (Boyd-Brewer and McCaffrey, 2004; Park et al., 2019). Current clinical evidence substantiates the therapeutic value of VAT across various chronic pain conditions, including low back pain, fibromyalgia, and postherpetic neuralgia. A 12-week prospective pilot study involving 23 patients with chronic low back pain revealed that sinusoidal vibration (16–160 Hz) resulted in a mean reduction of 3.5 points on the VAS and 13.5 points on the Pain Disability Index (PDI), with 65% and 52% of participants achieving clinically significant improvements in pain relief and physical function, respectively (Lim et al., 2018). Notably, a low-frequency stimulus of 40 Hz has been identified as a highly effective functional frequency for modulating both pain perception and motor impairment. However, the application of VAT in acute pain remains in its nascent stages, with existing research primarily focused on perioperative settings. (Ahn et al., 2024; Boyd-Brewer and McCaffrey, 2004; Park et al., 2019; Wang et al., 2021). Furthermore, the clinical implementation of VAT is characterized by significant heterogeneity regarding treatment duration and anatomical stimulation sites, and a consensus on optimal parameters has yet to be established. At present, VAT is frequently categorized as a complementary modality to MT. While their synergistic application is common, robust evidence isolating the independent efficacy of VAT in pain management is currently lacking. Consequently, future research should prioritize large-scale RCTs. By comparing monomodal VAT protocols with multimodal intervention groups, such studies will be essential to further elucidate the clinical utility of VAT within multimodal analgesic strategies.

## 5 Digital paradigms for acoustic stimulation in multimodal analgesia: immersive interactive technologies

In immersive interactive technologies, acoustic input is generally incorporated as part of a multisensory analgesic environment rather than as an isolated acoustic intervention. In the virtual reality (VR), augmented reality (AR), and mixed reality (MR) studies reviewed in this section, auditory components are mainly delivered as background music, environmental or naturalistic sounds, game-related sound effects, verbal narration, guided-relaxation audio, or procedure-related sounds embedded within visual and interactive scenarios. These acoustic elements may contribute to attentional engagement, emotional regulation, procedural familiarity, and the sense of presence within multimodal analgesic paradigms.

### 5.1 Virtual reality (VR)

Virtual reality is defined as a non-pharmacological clinical intervention that utilizes wearable displays, such as head-mounted displays (HMDs), to immerse users in a three-dimensional digital environment, thereby blocking sensory input from the physical world. Based on patient demographics, cognitive profiles, pain phenotypes, and therapeutic objectives, VR interventions are categorized into distinct modalities, including distraction therapy, exposure therapy, guided relaxation, and biofeedback-assisted therapy (Kalsotra et al., 2025).

In VR-based analgesic studies, auditory input is commonly delivered through headphones, earphones, or built-in audio systems and is integrated with visual immersion and interactive tasks. Depending on the intervention design, the acoustic component may include game sounds, calming background music, naturalistic environmental sounds, or guided-relaxation audio.

Clinical evidence increasingly underscores the broad potential of VR in multimodal pain management. In scenarios involving acute pain, such as traumatic stress and invasive procedures, VR exhibits potent immediate analgesic and anxiolytic effects. A single-center, dual-setting RCT demonstrated that passive immersive VR distraction significantly reduced both the Revised Faces Pain Scale (FPS-R) and visual analog anxiety scores in patients undergoing peripheral intravenous catheterization (PIVC) (*P* < 0.001). (Gold et al., 2021). Similarly, in pediatric burn patients, Xiang et al. reported that the active interactive VR distraction group exhibited significantly lower self-reported global pain scores (VAS, 0–100) compared to the standard care group [24.9 (95% CI: 12.2–37.6) vs. 47.1 (95% CI: 32.1–62.2); *P* = 0.02] (Xiang et al., 2021). Notably, the robust analgesic efficacy of VR persists even in patients with chronic conditions requiring frequent invasive interventions, suggesting potential universal applicability (Gold et al., 2021).

Recently, the clinical value of VR in chronic pain management has gained significant traction. Emerging neuroscientific research indicates that VR can inhibit central sensitization—a pivotal mechanism in the transition from acute to chronic pain—a finding that has been corroborated in clinical practice (Shi and Wu, 2023). In a RCT involving 128 hospitalized cancer patients, Groninger et al. (2024) observed that a 10-min immersive VR guided-relaxation intervention significantly reduced pain, with superior immediate analgesic efficacy compared to two-dimensional guided imagery (*P* = 0.03), and demonstrated a sustained analgesic post-effect for up to 24 h (*P* = 0.004). In chronic conditions such as low back pain, cervical pain, migraines, and fibromyalgia, VR frequently serves as a digital adjunct to Cognitive Behavioral Therapy (CBT). It enhances analgesic outcomes by facilitating the integration of sensory, cognitive, and affective dimensions (Chuan et al., 2021; Ding M. E. et al., 2025; Groninger et al., 2024; Guo et al., 2023). However, empirical exploration regarding common chronic conditions, such as diabetic neuropathic pain, remains sparse. Furthermore, the co-administration of VR with pharmacological analgesics has demonstrated significant synergistic value, potentially mitigating drug-related adverse effects (Shi and Wu, 2023). Future research should prioritize comparative analyses of various VR modalities tailored to specific pain subtypes. Moreover, identifying optimal therapeutic parameters for distinct patient populations will be essential for developing precision-based, individualized pain management protocols.

### 5.2 Augmented reality (AR)

Distinct from the fully synthetic environments constructed by VR, AR integrates computer-generated digital elements into the tangible physical environment, thereby enhancing the perceptual connection with the real world (Fleet et al., 2025; Kanschik et al., 2023; Rizzo et al., 2023). As an innovative non-pharmacological intervention, AR has historically been utilized for medical education and clinical training; however, its application within therapeutic domains such as pain management is currently in a nascent stage (Yeung et al., 2021). In AR-based analgesic applications, acoustic input is most often used as verbal narration, procedural explanation, or instructional guidance accompanying visual augmentation. This auditory information may support patient education, reduce procedural uncertainty, and modulate anxiety-related pain amplification. In the perioperative care, AR has been shown to reduce STAI scores in ambulatory surgery patients, with over 70% of participants in the AR group demonstrating high subjective adherence and recommendation intent (Kanschik et al., 2023). A preliminary study involving 20 pediatric oncology patients further demonstrated superior quality-of-life scores in the AR group compared to the control group (*P* < 0.001) (Prahm et al., 2025). By leveraging its immersive capabilities, AR can mitigate the “pain-anxiety” vicious cycle prevalent in perioperative settings by enabling patients to cognitively preempt real surgical scenarios, thereby alleviating psychological distress (Yeung et al., 2021).

Furthermore, by providing visual feedback-driven autonomous control, AR exhibits unique advantages in the management of phantom limb pain (PLP). Thøgersen et al. (2020) reported that AR significantly reduces pain intensity and maladaptive cortical reorganization in PLP patients, suggesting that its capacity for neural circuit remodeling may surpass that of VR. Based on these findings, it is hypothesized that AR holds substantial therapeutic potential for other forms of neuropathic pain, although the evidence base for this application requires further rigorous validation. Overall, given its unique “reality-enhancing” characteristics and potential for promoting neuroplasticity, AR is poised to become a focal point of research in the next generation of pain management strategies.

### 5.3 Mixed reality (MR)

Mixed reality represents an advanced integration of VR and AR, transcending simple digital overlays by facilitating responsive, real-time interactions between virtual elements and physical objects through sensorimotor integration (Prahm et al., 2025). In MR-based pain interventions, the currently reported therapeutic components are primarily visual-motor feedback, embodiment, agency, and sensorimotor interaction. Acoustic feedback, when included, is generally not described as a distinct intervention component, although spatial or interactive audio could theoretically reinforce realism and multisensory integration. Currently, the clinical potential of MR is most pronounced in the management of complex chronic pain conditions, particularly PLP. By seamlessly synchronizing haptic feedback with high-fidelity visual stimuli, MR offers a level of immersion and agency that may surpass the efficacy of traditional mirror therapy (Matthie et al., 2022; Prahm et al., 2025). Despite these theoretical advantages, the widespread clinical adoption of MR remains constrained. Significant barriers include high technical thresholds, substantial hardware costs, and the prohibitive complexity of the requisite interaction algorithms.

Overall, the available studies indicate that acoustic information is often embedded within immersive analgesic protocols but is seldom reported with sufficient methodological detail. Future VR, AR, and MR studies should more explicitly describe the type, delivery mode, duration, intensity, and spatial characteristics of acoustic input to clarify its contribution to multimodal analgesia.

## 6 Discussion

### 6.1 Imbalance in subtype distribution

While the advantages of AS—including high efficacy, non-invasiveness, safety, and cost-effectiveness—are widely acknowledged, existing evidence-based data exhibit a significant imbalance in subtype distribution. Current research is predominantly concentrated on perioperative pain, fibromyalgia, chronic back pain, and phantom limb pain. In contrast, clinical evidence regarding neuropathic pain (NeP) subtypes, such as postherpetic neuralgia (PHN) and diabetic peripheral neuropathy (DPN), remains comparatively sparse (Table 1) (Cata et al., 2022; Chanda and Levitin, 2013; Chuan et al., 2021; Cohen et al., 2021; Ding S. et al., 2025; Gkolias et al., 2020; Gold et al., 2021; Guo et al., 2023; Honzel et al., 2019; Inayama et al., 2022; Kahraman et al., 2020; Kalsotra et al., 2025; Koelsch and Bradt, 2025; Muzzi et al., 2021; Schmid et al., 2020; Shah et al., 2017; Shi and Wu, 2023; Soo et al., 2016; Thøgersen et al., 2020). Critically, patients with neuropathic pain often present with refractory and intractable chronic symptoms complicated by severe emotional distress. The clinical remission rate for these populations remains suboptimal, typically ranging between 30% and 40% (Attal et al., 2023). AS offers a promising therapeutic avenue for these difficult-to-treat cases; it not only attenuates the sensory and affective dimensions of pain through diverse neurobiological pathways but also effectively disrupts the “pain-anxiety-insomnia” vicious cycle. By acting on multiple targets within nociceptive neural circuits, AS may facilitate the reversal of maladaptive neuroplasticity, ultimately enhancing both quality of life and psychological wellbeing. Consequently, future research should prioritize the clinical utility of AS in neuropathic pain management, aiming to refine multimodal analgesic strategies tailored to diverse pain phenotypes.

**TABLE 1:** Summary of key clinical studies on acoustic stimulation interventions for pain management.

Category	References	Participants	Study design	Sample size	Conclusions
MT	Nowak et al., 2020	Adults, elective surgery (GA, 1–3 h)	RCT	*n* = 400	Therapeutic verbal suggestions delivered during general anesthesia have been shown to significantly reduce postoperative opioid requirements and alleviate pain, representing a safe, cost-effective, and non-pharmacological clinical intervention.
MT	Bradt et al., 2024	Patients with chronic cancer-related pain	RCT	*n* = 92	MT not only alleviates pain but also enhances patients’ self-efficacy by fostering music-assisted self-management strategies, thereby optimizing long-term pain management outcomes in patients with advanced cancer.
MT	Alparslan et al., 2016	Patients with fibromyalgia	RCT	*n* = 37	MT demonstrates significant efficacy in the management of fibromyalgia-related pain. Given its cost-effectiveness, ease of implementation, and favorable safety profile, it is recommended as a viable non-pharmacological nursing intervention within the clinical management framework for fibromyalgia.
MT	Torlak et al., 2025	Patients with chronic neck pain	RCT	*n* = 40	The integration of passive music listening as an adjunct to standard physical therapy significantly enhances pain relief in patients with chronic neck pain.
NS	Kutenai et al., 2023	Burn patients (ICU setting)	RCT	*n* = 60	Both Benson’s relaxation technique and exposure to NS serve as effective non-pharmacological interventions for managing pain and anxiety in patients within the burn ICU.
NS	Parandavar et al., 2025	Pregnant women undergoing cesarean section (C-section)	RCT	*n* = 92	The application of NS during cesarean section under spinal anesthesia significantly improves neonatal Apgar scores, representing an effective and clinically promising intervention for enhancing neonatal outcomes.
NS	Lepping et al., 2022	Patients with fibromyalgia	Double-blind, randomized pilot study	*n* = 10	MT may exert superior physiological relaxation and analgesic effects compared to NS by more effectively enhancing vagal tone.
Noise	Lin et al., 2024; Kolhe et al., 2024	Pediatric patients undergoing primary tooth extraction	RCT	*n* = 40	Audio distraction is an innovative, non-invasive, and effective behavior management technique that significantly mitigates dental anxiety in pediatric patients.
BB	Schmid et al., 2020; Ölçücü et al., 2021	Male patients undergoing cystoscopy and ureteral stent removal	RCT	*n* = 352	BB significantly reduce anxiety levels and pain scores in male patients undergoing cystoscopy and ureteral stent removal.
BB	Tani et al., 2021	Elderly patients undergoing total knee arthroplasty (TKA)	RCT	*n* = 40	Preoperative BB stimulation serves as an effective non-pharmacological intervention for elderly patients undergoing total knee arthroplasty, reducing morphine consumption and promoting postoperative recovery.
BB	Halpin et al., 2025; Schmid et al., 2020	Pediatric patients (infraumbilical surgery)	RCT	*n* = 49	Brainwave entrainment effectively reduces the propofol infusion rate required for sedation in pediatric patients undergoing regional anesthesia.
BB	Gkolias et al., 2020	Patients with chronic pain	RCT	*n* = 21	Listening to θ-frequency BB effectively alleviates pain intensity, reduces dependence on analgesics, and provides long-term stress relief for chronic pain patients.
VAT	Lim et al., 2018	Patients with chronic low back pain (CLBP)	Pilot study	*n* = 23	Passive low-frequency acoustic VAT applied to the limbs has demonstrated significant efficacy in alleviating pain and improving functional status in patients with chronic back pain.
VAT	Wang et al., 2021	Patients with postherpetic neuralgia (PHN)	Case reports	*n* = 1	Low-frequency acoustic stimulation, as a complementary and alternative therapy, shows significant efficacy in the management of refractory neuropathic pain.
VR	Gold et al., 2021	Patients undergoing peripheral intravenous catheterization (PIVC)	RCT	*n* = 107	Patients receiving VR intervention during PIVC exhibit significantly lower anxiety and pain scores compared to those receiving standard care.
AR	Cata et al., 2022	Pediatric patients undergoing oncological surgery	Feasibility RCT	*n* = 20	Implementing an AR-based treasure hunt game is a feasible intervention for children in pediatric oncology post-care settings.
AR	Thøgersen et al., 2020	Patients with phantom limb pain (PLP)	Proof-of-concept study	*n* = 7	Personalized AR visual feedback training demonstrates significant analgesic and neuro-reparative effects in patients with PLP characterized by phantom limb shortening.
MR	Prahm et al., 2025	Patients with PLP vs. healthy controls (HC)	Clinical usability evaluation	*n* = 18	PhantomAR validates that MR-assisted dynamic bilateral interactive training significantly alleviates PLP and effectively enhances patients’ sense of agency over the residual limb.

MT, music therapy; NS, natural sounds; BB, binaural beats; VAT, vibroacoustic therapy; VR, virtual reality; AR, augmented reality; MR, mixed reality; GA, general anesthesia; ICU, Intensive Care Unit; RCT, randomized controlled trial; TKA, total knee arthroplasty; CLBP, chronic low back pain; PHN, postherpetic neuralgia; PIVC, peripheral intravenous catheterization; PLP, phantom limb pain; HC, healthy controls.

### 6.2 Standardization of intervention paradigms and methodological rigor

In clinical settings, a standardized consensus regarding the optimal stimulus parameters for AS—including duration, frequency, and intensity—has yet to be established. This significant parametric heterogeneity directly contributes to the high variability observed in current clinical evidence, complicating high-quality cross-sectional comparisons and systematic meta-analyses (Ding S. et al., 2025). Furthermore, historical research has disproportionately focused on MT and VR, frequently marginalizing the therapeutic potential of NS, noise, and IT. It is noteworthy that current research faces persistent challenges regarding the “placebo effect” and the lack of rigorous control designs. Particularly in immersive acoustic interventions, the unique multisensory (e.g., audiovisual) nature of the experience poses significant challenges to implementing strict blinding, which invites scrutiny regarding the veracity of their independent clinical efficacy.

Consequently, future research should transition beyond qualitative descriptions typical of opinion-based articles. Instead, investigators must adopt objective, quantitative tools for evidence-based evaluation, such as the Jadad scale and the Cochrane Risk of Bias (RoB) tool, to systematically assess research quality. Moving forward, the field should prioritize the standardization of methodological screening criteria. Future efforts must be directed toward conducting large-scale, high-quality RCTs aimed at elucidating the optimal treatment parameters and therapeutic windows for various AS modalities. Furthermore, rigorous evaluations of both the independent efficacy and synergetic effects of AS are essential to provide a robust evidence-based foundation for its standardized clinical implementation.

### 6.3 Confounding factors and personalized assessment

As a therapeutic modality rooted in multisensory integration, the efficacy of AS is highly susceptible to diverse confounding variables. A fundamental prerequisite for AS-mediated analgesia is the functional integrity and sensitivity of the patient’s sensory systems. For individuals with sensory hyperacusis (sensory hypersensitivity), even standard intensity levels may precipitate sensory overload, paradoxically exacerbating the perceived intensity of pain (Shi and Wu, 2023). Furthermore, the patient’s cognitive profile and subjective preferences represent critical determinants of the observed inter-individual variability in treatment outcomes (Arnold et al., 2024; Becker et al., 2025; Van der Valk Bouman et al., 2024). Exposure to acoustic environments perceived as aversive or discordant can trigger acute stress responses, potentially escalating anxiety and nociceptive signaling. Environmental factors also play a non-negligible role; ambient acoustic interference (background noise) can mask the intended analgesic signals, thereby neutralizing the therapeutic effect or inducing negative affect (Buxton et al., 2021). Consequently, it is imperative for technology developers to move beyond a “one-size-fits-all” approach and prioritize the creation of personalized content grounded in individual patient profiles. This transition represents both a significant technical hurdle and a strategic focus for the evolution of AS toward a precision medicine paradigm.

### 6.4 The evolution of digital technologies

With the emergence of digital health technologies, clinical pain management is transitioning from traditional, passive “auditory modulation” toward precise, “neuromodulation” paradigms centered on multisensory integration of acoustic information. Preliminary evidence suggests that emerging technologies, such as immersive interactive systems integrating acoustic stimuli, may involve more complex neurobiological mechanisms than previously understood. However, existing research primarily emphasizes clinical efficacy, with a notable scarcity of fundamental studies exploring the underlying mechanisms. This gap necessitates an integrative approach, incorporating neuroimaging, serum biomarkers, and multidimensional sensory assessments, to establish a robust, objective, and evidence-based framework for AS in pain management.

Despite the groundbreaking progress of immersive interactive technologies integrating acoustic information in pain relief, significant adverse effects—such as cybersickness and derealization—remain prevalent (Rizzo et al., 2023). Consequently, clinical implementation necessitates rigorous monitoring by healthcare professionals to mitigate potential risks. Furthermore, in patients with auditory or visual hypersensitivity, excessive sensory stimulation may paradoxically induce sympathetic hyperarousal, thereby exacerbating the pain experience (Shi and Wu, 2023). Therefore, the specific parameters of these interventions must be personalized and dynamically adjusted based on individual patient profiles.

Notably, while immersion, interactivity, and presence are the core determinants of the clinical analgesic value of immersive technologies, these elements currently lack systematic quantitative assessment. More importantly, existing analgesic protocols exhibit a distinct visual bias in methodological design, largely overlooking the synergistic potential of physical acoustic stimulation and spatial audio feedback. In fact, high-fidelity information and the integration of multisensory (i.e., audiovisual) channels may be critical for advancing immersive technologies toward standardized digital therapeutic paradigms. Currently, research remains dominated by VR, while the efficacy and safety of AR and MR in pain management remain inadequately characterized.

Notably, such immersive digital experiences significantly stimulate user imagination and curiosity, garnering high levels of acceptance among pediatric and adolescent populations (Logan et al., 2021). However, the current technological landscape is not without potential drawbacks, such as the risk of digital addiction. Investigators must prioritize the optimization of these digital tools while establishing comprehensive regulatory frameworks and risk assessment protocols to ensure the safety of digital analgesic interventions. Furthermore, the clinical and home-based implementation of these emerging technologies is currently constrained by high costs and environmental requirements, necessitating further innovation toward portable, cost-effective solutions. In summary, immersive reality interaction technologies—including VR, AR, and MR—integrated with complex spatial acoustic modulation, represent a promising new paradigm for digital intervention in the management of both acute and chronic pain.

### 6.5 Potential of isochronic tones (IT) as an emerging digital therapeutic

As a novel form of ABS in the field of neuromodulation, IT consist of discrete, repetitive, and highly regular sound pulses, with their mechanism of action likewise contingent upon neural entrainment. However, their physical characteristics differ fundamentally from those of BB. EEG-based analyses suggest that IT-induced cortical activation intensity may surpass that of both BB and white noise, indicating superior capacity for modulating synchronous neuronal activity (Dos Anjos et al., 2024). Nevertheless, the application of IT in pain management remains largely unexplored, with current literature predominantly focused on mental health and cognitive functioning (Dos Anjos et al., 2024; Jiao, 2025). Consequently, future research should prioritize establishing a clinical evidence base for IT in pain management, focusing on comparative analyses of efficacy and safety across diverse frequency bands. Such efforts are essential to provide the data-driven support required for the advancement of precise digital therapeutics for pain relief.

## 7 Conclusion

In summary, AS represents a promising non-pharmacological, non-invasive modality within multimodal analgesic strategies. While preliminary evidence demonstrates its clinical utility in the management of both acute and chronic pain, robust support from large-scale, high-quality RCTs remains essential. Future research must prioritize the comparative analysis of therapeutic efficacy across diverse intervention paradigms and stimulus parameters. Furthermore, the development of a quantifiable evaluation system—integrating neuroimaging, biochemical markers, and Quantitative Sensory Testing (QST)—is essential to elucidate the underlying mechanisms and monitor clinical outcomes objectively. Ultimately, bridging these gaps in methodological rigor and mechanistic understanding will be pivotal in driving the transformation of AS into precision-based, individualized analgesic protocols, offering a safer and more effective digital therapeutic option for diverse patient populations.
